# Endemicity and diversification of carbapenem-resistant *Acinetobacter baumannii* in an intensive care unit

**DOI:** 10.1016/j.lanwpc.2023.100780

**Published:** 2023-05-09

**Authors:** Emma L. Doughty, Haiyang Liu, Robert A. Moran, Xiaoting Hua, Xiaoliang Ba, Feng Guo, Xiangping Chen, Linghong Zhang, Mark Holmes, Willem van Schaik, Alan McNally, Yunsong Yu

**Affiliations:** a Institute of Microbiology and Infection, College of Medical and Dental Sciences, University of Birmingham, Birmingham, UK; b Department of Infectious Diseases, Sir Run Run Shaw Hospital, Zhejiang University School of Medicine, Hangzhou, Zhejiang, 310016, China; c Department of Veterinary Medicine, University of Cambridge, Cambridge, UK

**Keywords:** Carbapenem-resistant *Acinetobacter baumannii*, Intensive care, Genomic epidemiology, Infection prevention and control, Antimicrobial resistance, Horizontal gene transfer

## Abstract

**Background:**

Carbapenem-resistant *Acinetobacter baumannii* (CRAB) is a major public health concern globally. Often studied in the context of hospital outbreaks, little is known about the persistence and evolutionary dynamics of endemic CRAB populations.

**Methods:**

A three-month cross-sectional observational study was conducted in a 28-bed intensive care unit (ICU) in Hangzhou, China. A total of 5068 samples were collected from the hospital environment (n = 3985), patients (n = 964) and staff (n = 119). CRAB isolates were obtained from 10.5% of these samples (n = 532). All of these isolates, plus an additional 19 from clinical infections, were characterised through whole-genome sequencing.

**Findings:**

The ICU CRAB population was dominated by OXA-23-producing global clone 2 isolates (99.3% of all isolates) that could be divided into 20 distinct clusters, defined through genome sequencing. CRAB was persistently present in the ICU, driven by regular introductions of distinct clusters. The hospital environment was heavily contaminated, with CRAB isolated from bed units on 183/335 (54.6%) sampling occasions but from patients on only 72/299 (24.1%) occasions. CRAB was spread to adjacent bed units and rooms, and following re-location of patients within the ICU. We also observed three horizontal gene transfer events between CRAB strains in the ICU, involving three different plasmids.

**Interpretation:**

The epidemiology of CRAB in this setting contrasted with previously described clonal outbreaks in high-income countries, highlighting the importance of environmental CRAB reservoirs in ICU epidemiology and the unique challenges in containing the spread of CRAB in ICUs where this important multidrug-resistant pathogen is endemic.

**Funding:**

This work was undertaken as part of the DETECTIVE research project funded by the 10.13039/501100000265Medical Research Council (MR/S013660/1), 10.13039/501100001809National Natural Science Foundation of China (81861138054, 32011530116, 31970128, 31770142), Zhejiang Province Medical Platform Backbone Talent Plan (2020RC075), and the 10.13039/501100012166National Key Research and Development Program of China grant (2018YFE0102100). W.v.S was also supported by a Wolfson Research Merit Award (WM160092).

**Research in context**

**Evidence before this study**

A search of NCBI PubMed with the term “carbapenem resistant *Acinetobacter baumannii* ICU” returned 585 results (search conducted July 19th, 2021). The vast majority are reports of small localised outbreaks characterising clinical isolates only. We found three publications reporting room closure and deep cleaning interventions that reduced clinical CRAB infections. However, we found no genome-level investigations of the combination of colonisation, environmental contamination and clinical infections in a hospital setting.

**Added value of this study**

We conducted a comprehensive genome-level observational study of the prevalence and movement of CRAB in an ICU in a high-prevalence setting. By utilising intense sampling of the ICU environment and monitoring patients, we show the extent of CRAB colonisation, contamination and transmission within the ICU. Our data also shows the frequency with which CRAB is introduced into the ICU and how quickly CRAB populations adapt to the ICU setting and share mobile genetic elements via horizontal gene transfer.

**Implications of all the available evidence**

The high prevalence, multi-clonal nature and high degree of genome plasticity of CRAB in this ICU highlights the urgent need for targeted infection prevention and control measures in high-prevalence settings to stem the global spread of this multidrug-resistant nosocomial pathogen.

## Introduction

Antibiotic-resistant nosocomial infections are a major threat to global public health. *A. baumannii* is a Gram-negative coccobacillus that causes severe disease including pneumonia, urinary tract infection, bacteraemia, meningitis, or skin and soft tissue infections, particularly in hospitalised patients.^1^ Carbapenem-resistant *A. baumannii* (CRAB) are found worldwide and are often only sensitive to tigecycline and polymyxins,^2^ severely limiting treatment options. This prompted the World Health Organisation to designate CRAB a priority organism for which novel therapeutics are urgently required.^3^ In the absence of new therapeutic agents, effective CRAB infection prevention and control (IPC) strategies are important to limit the morbidity and mortality it causes in hospitals.^4^ To this end, it is crucial to develop a thorough understanding of the persistence, transmission and evolution of CRAB populations in nosocomial environments.

*A. baumannii* can persist for prolonged periods on hospital surfaces and medical equipment, and colonise patients within 48 h of admission.^1^ Asymptomatic cutaneous, pharyngeal and gastrointestinal carriage has often been associated with heavy contamination of patients’ immediate environments.^1^ Transmission is thought to be facilitated by hospital staff, shared equipment, airflow and plumbing.^1^ Outbreaks of *A. baumannii* can prove intractable, requiring interventions or changes to infrastructure that impose clinical, logistical, and financial burdens.^5^

Globally, CRAB populations are dominated by two clones, Global Clone 1 (GC1) and Global Clone 2 (GC2), which correspond to ST1 and ST2, respectively, in the Pasteur MLST scheme.^2^ Carbapenem resistance in GC1 and GC2 isolates is most commonly conferred by the *bla*_OXA-23_ carbapenemase gene, located in IS*Aba1*-bounded composite transposons Tn*2006* or Tn*2009* that are usually inserted in the chromosome.^2^ In other cases, *bla*_OXA-23_ or metallo-β-lactamase genes such as *bla*_NDM-1_ are plasmid-borne.^2^ *Acinetobacter* plasmids are genus-specific and have been typed according to the relatedness of their replication initiation proteins.^6^ However, given until recently there were no publicly-available tools or databases for rapidly identifying and typing *Acinetobacter* plasmids, they are often overlooked in genomic studies.

Epidemics of CRAB often occur in high-income countries after a breach of IPC procedures, introducing and spreading a single clone of CRAB within the hospital.^7^ Such clonal outbreaks are typically resolved after outbreak investigation and targeted interventions. Recent studies have demonstrated the utility of whole-genome sequencing (WGS) for high-resolution characterisation of such single-centre outbreaks or populations.^4^^,^^8^, ^9^, ^10^ Endemic hospital *A. baumannii* populations, however, can be composed of multiple phylogenetic clusters.^11^^,^^12^ WGS has been employed to assess multi-centre CRAB populations, revealing that individual hospitals harbour multiple clusters and that these may be found across multiple hospitals.^13^^,^^14^ These studies have focused on isolates derived from clinical or patient screening specimens. Given its persistence on surfaces, environmental isolates must also be considered in order to understand the distribution and dynamics of CRAB within individual hospitals in endemic countries. A high-resolution assessment of CRAB strain dissemination, cluster evolution, and horizontal gene transfer dynamics requires deep-sampling and WGS of CRAB in a single setting and this provided the motivation for undertaking this study.

Here we describe a cross-sectional observational study of CRAB in an ICU in Hangzhou, China. Over a three-month period, a deep-sampling approach targeted patients, the hospital environment and hospital staff. Isolates were whole-genome sequenced and high-resolution approaches were used to investigate population structure, dynamics of strain movement and dissemination, and horizontal gene transfer events within the ICU.

## Methods

Methods are outlined briefly here. Full details are provided in the Supplementary materials.

### Consent and research ethics

Ethical approval and informed consent were obtained by the Sir Run Run Shaw Hospital (SRRSH) Zhejiang University local ethics committee (approval number 20190802-1). When patients were admitted to the inpatient ward (all departments, including the ICU), hospital staff spoke with them and/or their family members to obtain informed consent for sample collection. Electronic informed consent documents were collected and stored. This work was part of a study registered as a clinical trial with ClinicalTrials.gov (NCT04310722). The participation of hospital staff was voluntary.

### Study design and sample collection

We conducted a cross-sectional observational study in a 28-bed ICU in SRRSH, Hangzhou, China from August to October 2019 (Figure S1). We planned for patients to be sampled at the beginning of the study or on admission to the ward and weekly thereafter, so long as sampling was not perceived to be medically detrimental. Patient samples were routinely collected from oral and rectal swabs, and from nasogastric, nasojejunal, endotracheal, or tracheostomy tube swabs when present. Clinical samples were taken as part of normal medical practice, and all resulting CRAB isolates were collected. Within bed units, equipment and surfaces were swabbed weekly while the surfaces of sinks in patient rooms were sampled fortnightly. Equipment, surfaces, and sinks in communal areas outside patient rooms were sampled monthly. Staff rectal swabs or stool samples were provided in the first week of each month. An overview of the sampling and isolation strategy is presented in Figure S2.

Family members could access the ICU only at a specific time in the afternoon of each day, and were required to wear protective suits. Family members did not provide nursing care. Nurses were responsible for bathing all patients.

### Sample processing and DNA sequencing

Samples were cultured on Acinetobacter CHROMagar plates containing 2 μg/mL meropenem. DNA was extracted from one isolate per culture-positive sample and Illumina sequenced. A subset of 60 isolates were selected based on unique phylogenetic clustering, antibiotic resistance genes and plasmids for long-read sequencing with the Oxford Nanopore GridION.

### Bioinformatic analysis

Sequence reads were trimmed, assembled and assessed for quality. MLST (https://github.com/tseemann/mlst) was used to determine multi-locus sequence types with the Pasteur and Oxford typing schemes.^15^^,^^16^ Typing of capsular polysaccharide (KL) and lipooligosaccharide outer core (OCL) synthesis loci was conducted with Kaptive.^17^ AMRFinder was used to identify antimicrobial resistance genes.^18^ A searchable database of all contigs from all Illumina genomes in this collection was constructed and queried with sequences listed in Table S10 using standalone BLAST.^19^

Snippy v4.4.5 (https://github.com/tseemann/snippy) was used to align Illumina reads against a corresponding reference hybrid assembly and generate a core genome alignment. Polymorphic sites were extracted with Gubbins v2.4.0 excluding those that were predicted to occur via recombination.^20^ Phylogenies were constructed from these polymorphic sites using RaxML v8.2.12 with the GTRGAMMA model and autoMRE rapid bootstopping.^21^ The GC2/ST187 population was partitioned into clusters using FastBAPS.^22^ Divergence dating was undertaken with the least-squares method implemented by IQTree v2.0.3, using the previously generated RaxML tree, Gubbins fasta file, and a GTR + G model.^23^^,^^24^ SNP-distances were calculated from the Gubbins-filtered polymorphic sites file using SNP-dists 0.6.3 (https://github.com/tseemann/snp-dists).

Plasmid replicons were initially typed using a custom database (see https://www.medrxiv.org/content/10.1101/2022.05.19.22275186v1 for details), before they were re-typed according to the latest typing scheme, which was published while this manuscript was under review.^25^

### Role of the funding source

The funders had no role in study design, data analysis, or manuscript preparation. The authors were not precluded from accessing data in the study, and accept responsibility to submit for publication.

## Results

### Abundance of CRAB in patients and the patient environment of the ICU

Over the three-month study, a total of 140 patients (102 male; 38 female; median age 80 years; interquartile range [IQR] = 63.2–85.6) were sampled. The median length of stay from admission to discharge or the end of the study was six days (IQR: 3–15 days). Samples were taken each Tuesday, except during week 9 due to a national holiday and patients were screened within the first two full days after admission when possible. “Bed units”, each defined as the environmental sites of each bed and its associated equipment (Figure S1), were sampled on 335/336 (99.7%) planned sampling occasions. Patients were sampled on 299/318 (94.0%) of planned occasions. Fifteen patients stayed in the ICU but were never sampled.

In total, 5068 samples were collected (Table S1; excluding 19 clinical specimens). CRAB was isolated from 532 samples (10.5%): 432/3985 (10.8%) environmental samples and 100/964 (10.4%) patient-screening samples. None of the 119 staff samples were CRAB-positive. CRAB was isolated from the bed unit and the patient in the bed on 183/316 (57.9%) occasions. CRAB was isolated more frequently from bed unit environments (183/335, 54.6%) than patients (72/299, 24.1%). Bed units yielded more environmental isolates (428/3095, 13.8%) than communal areas outside bed units (5/80 samples, 6.3%). Within bed units, samples from ventilators (80/287 samples, 27.9%) and dispensing trolleys (10/39 samples, 25.6%) were most likely to yield CRAB (Table S1). Almost a third (35/125, 28.0%) of patients screened were CRAB-positive during the study, with most of the positive samples originating from oral (32/254, 12.6%) or rectal (41/289, 14.2%) swabs from 19 to 22 patients, respectively. Additionally, 19 diagnostic clinical isolates were collected from 12 patients.

MICs for six antibiotics were determined for all 551 isolates (Supplementary dataset 1). This confirmed that all isolates were resistant to imipenem and meropenem. All isolates were resistant to ciprofloxacin but sensitive to polymyxin B and tigecycline, and 274 isolates (49.7%) were resistant to amikacin.

### GC2 dominated the CRAB population, characterised by a number of clusters of low diversity isolates

**Fig. 1:**
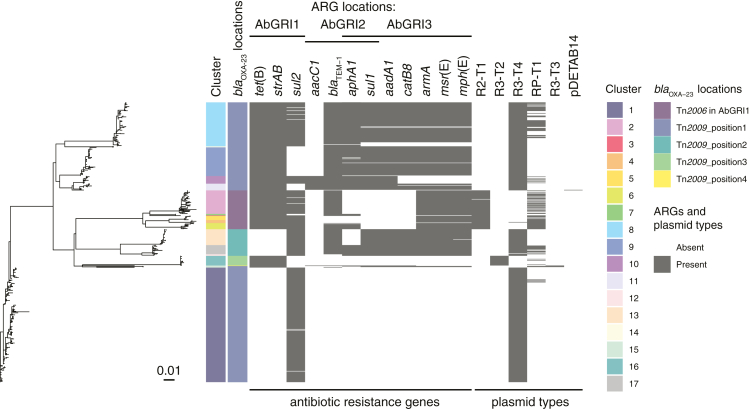
**Characterisation and comparison of GC2/ST187 CRAB found in the ICU.** Evolutionary relationships of the 547 GC2/ST187 isolates from the ICU; coloured tracks adjacent to the phylogenetic tree tip show the cluster designation, Oxford MLST types, KL types and different positions of *bla*_*OXA-23*_ genes; presence of each antibiotic resistance gene and plasmid type is shown in grey and their absence is in white. pDETAB14 represents a novel plasmid type that was found in a single C11 isolate.

We performed WGS analysis on all 551 isolates and investigated CRAB diversity in the ICU. The majority of isolates, 543 (98.5%), were classified according to the Pasteur MLST scheme as GC2, and four (0.7%) were ST187 (Fig. 1), which differed from GC2 by a single nucleotide polymorphism (SNP) in the *rpoB* locus. The remainder were ST46 (n = 2), ST138 (n = 1) and a novel ST (n = 1) that was designated ST1554 after submission to PubMLST. The ST138 and ST1554 isolates have been described previously.^26^^,^^27^ All GC2/ST187 isolates carried the *bla*_OXA-23_ carbapenemase gene in either Tn*2006* or Tn*2009* inserted in one of five chromosomal positions (Fig. 1). Other acquired antibiotic resistance genes found in GC2/ST187 isolates conferred resistance to sulphonamides, aminoglycosides, tetracycline or macrolides and were in variations of the AbGRI1, AbGRI2 and AbGRI3 chromosomal resistance islands (Fig. 1). IS*26*-mediated deletion events have been responsible for the loss of resistance genes from these islands (Fig. 1). Amikacin resistance was strongly associated with the presence of the *armA* aminoglycoside resistance methylase gene in AbGRI3. All 271 *armA*-containing isolates were amikacin resistant, but the remaining three amikacin-resistant isolates did not contain *armA*.

Diversity of the GC2/ST187 population was explored in terms of Oxford MLST and typing of the capsular polysaccharide (KL) and lipooligosaccharide outer core (OCL) synthesis loci. Amongst the 547 isolates, eight Oxford STs and ten KL were identified (Fig. 1). All isolates were OCL1. K loci were assigned with variable match confidence levels and assignment problems were reported for some isolates. In many cases Oxford ST and/or KL profiles were incongruent with the phylogenetic population structure (Fig. 1). Both KL7 and KL28 occurred within the same phylogenetic clade while ST_OX_136, ST_OX_208, ST_OX_540, KL2, KL7, KL9, KL28 and KL77 were assigned to isolates from multiple polyphyletic clades. Neither Oxford MLST nor K/OCL typing were able to adequately distinguish subpopulations of GC2/ST187 circulating in the ICU for epidemiological typing purposes.

Phylogenetic and population genetic analysis of the GC2/ST187 isolates revealed that this population had a maximum of 194 core-genome SNPs between isolates. These were used to partition the population into 17 distinct GC2/ST187 clusters (C1–C17) using FastBAPS (Fig. 1). Published mutation rates for GC2 *A. baumannii* estimate a rate of approximately 24 substitutions per year for within host evolution and approximately 10 substitutions per year for GC2 spreading between hosts.^10^^,^^28^ The minimum SNP distance between our 17 clusters ranged from 12 to 117 SNPs indicating that the diversity seen across our GC2 isolates has not accumulated within the ICU from a single source introduction during the sampling period (Table S2). Dating analysis using IQTree supported this assertion (Table S3), with the most recent common ancestors (MRCA) between clusters predicted to occur prior to the start of this study (from 1992 to 2016). Combining these data with published mutation rates and IQTree dating shows that these clusters did not arise from one another over the course of this study. Rather, each of these clusters was introduced separately or circulating independently within the ICU during our sampling period. The median within-cluster SNP distance across the 17 clusters ranged from 0 to 8 SNPs with the exception of C15 which has a within-cluster median SNP distance of 76 SNPs (Table S3).

Of 53 patients screened within 48 h of ICU admission, four (7.5%) yielded CRAB from oral, rectal or indwelling tube swabs. Three of these samples represented the first or only appearance of ST138, C3 and C9, strongly suggesting that they were introduced to the ICU with their respective patients. The fourth patient also yielded C9, which had not been detected in the ICU for six weeks prior to their admission, suggesting that C9 was introduced to the ICU on two occasions by two different patients. Additionally, three patients yielded CRAB from sputum samples collected for clinical purposes within 48 h of ICU admission. Two of these represented the first appearances of C14 and C16. Thus, at least five CRAB clusters appear to have been introduced to the ICU with patients (Table S4).

### Extensive strain spread in the ICU and acquisition by patients was driven by environmental contamination

Six CRAB clusters (C3, C12, C14, and ST46, ST138 and ST1554), each with a maximum of three isolates and associated with a single patient were considered sporadic. The remaining 14 clusters were found in between two and 49 different patients or their bed unit environments (Table S5). For these clusters, the median number of cgSNPs was between 0 and 8 SNPs, and the distribution of small cgSNP frequencies around a mode (Figure S3) was clearly indicative of their recent and ongoing spread within the ICU.

**Fig. 2:**
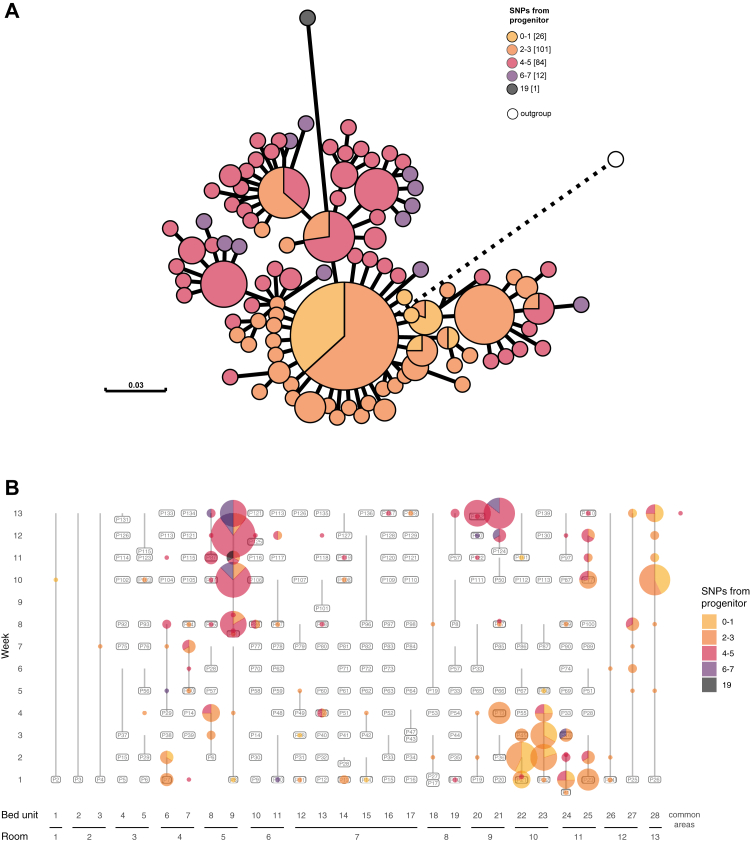
**Introduction and spread of cluster 1 within the ICU as measured by whole genome SNPs.** A) Maximum-likelihood core-genome phylogeny of all 224 cluster 1 (C1) isolates using a cluster 11 isolate (DETAB-E47) as an outgroup. Visualised in GrapeTree and rooted on the outgroup, shown in white. Colours show the number of SNPs from the most basal C1 isolate, with up to 7 cgSNPs in the nosocomial population, and 19 cgSNPs in the most distal isolate. B) Spatiotemporal distribution of C1 isolates, coloured as in panel A.

C1, represented by 224 isolates, was isolated in each week of the study period, and at least once from each of the patient rooms in the ICU. This dominant and seemingly endemic cluster provided an opportunity to examine the dynamics of a clonal population that persisted in the ICU environment for the entirety of the study period. A maximum-likelihood core-genome phylogeny revealed that all but one isolate in C1 differed by ≤7 SNPs from the most basal isolate (Fig. 2A). The distribution of C1 throughout the study indicates it persisted and spread within the ICU (Fig. 2B) and was introduced on multiple occasions from a broader nosocomial population. For the first seven weeks, the C1 population was dominated by isolates that differed by ≤3 SNPs from the basal isolate (orange shades in Fig. 2), while isolates that differed by 4–7 SNPs (pink/purple shades in Fig. 2) appeared sporadically. In the final six weeks of the study, isolates 4–7 SNPs different from the basal isolate dominated the population, centred around the environment of room 5 (Fig. 2B). The simultaneous isolation of different sub-populations of C1 from the same bed units suggest that this cluster persisted and diversified in this hospital for an extended period of time and was introduced into the ICU on multiple occasions.

**Fig. 3:**
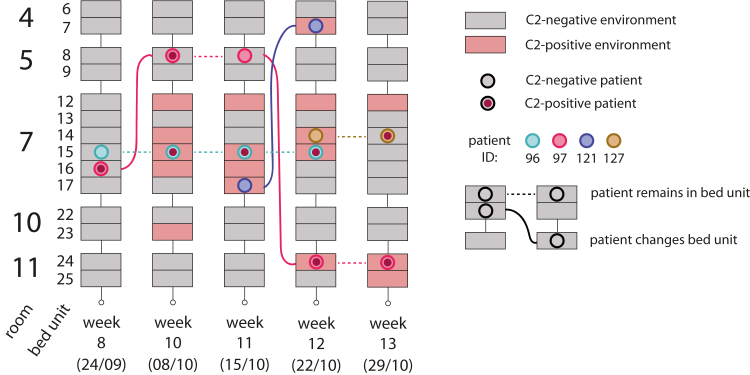
**Spatiotemporal distribution of cluster 2 isolates in the ICU.** Schematic showing the distribution of all GC2 cluster 2 (C2) isolates in rooms 4, 5, 7, 10 and 11 over weeks 8–13 of the study. These were the only instances where C2 was isolated over the course of the study. Sampling dates (day/month) are shown in brackets. Each bed unit is shown as a rectangle, shaded red when C2 was isolated from the environment and grey when it was not. Patients are shown as coloured circles, with a red fill indicating the presence of C2 in patient-screening samples. Dashed horizontal lines represent a patient's continued presence in a bed unit while solid lines show the relocation of patients between sampling dates.

C2 was introduced to the ICU during the study period by a single patient in week 8, facilitating an examination of its subsequent dissemination dynamics (Fig. 3; Table S4). After the first isolation of C2 from a rectal swab from patient 97 in week 8, C2 was isolated from the environment and other patients staying in the same room for the remainder of the study. Movement of patient 97 and a patient whose bed unit had been colonized by C2 then contributed to the spread of this cluster in the ICU.

**Fig. 4:**
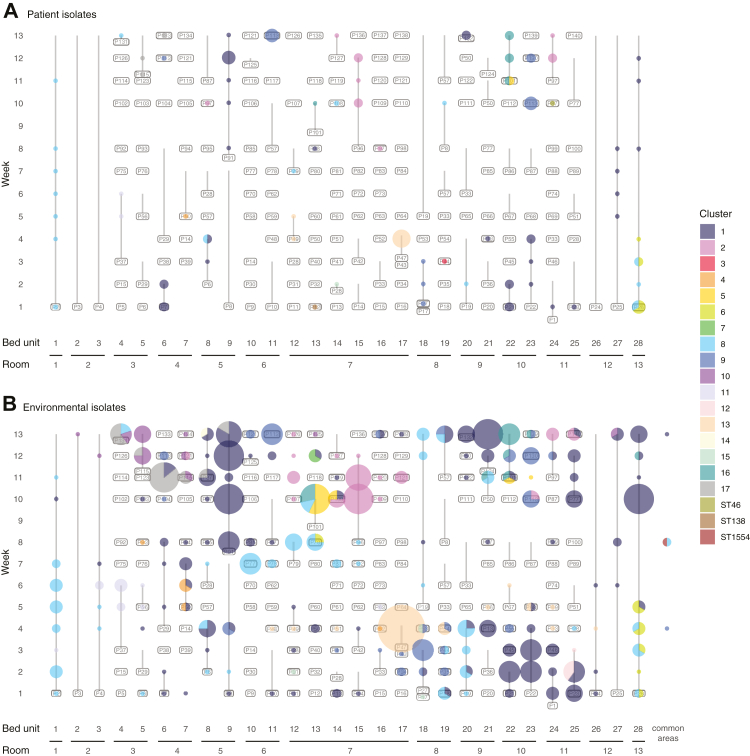
**Diversity and distribution of CRAB isolates within the ICU.** Distribution of isolates from each CRAB cluster (Panel A: isolates from patient samples, panel B: isolates from environmental samples). Bed unit numbers are indicated across the x-axis and reflect the spatial arrangement of beds in the ICU (Figure S1); isolates from common areas are grouped together but may be from spatially distinct locations; the vertical axis shows the week of sampling; labels show the ID of the patient occupying the bed with vertical lines extending from the date of first to last sample associated with the patient; size of coloured bubbles reflects the number of CRAB isolates found in the patient and their bed unit on each date of sampling; the colour of bubbles corresponds to the CRAB cluster; multi-coloured bubbles are split as a pie chart to reflect the proportion of isolates from each cluster.

The spatiotemporal distribution of clusters was used to assess potential patterns of CRAB movement and persistence in the ICU (Fig. 4). Of 119 occasions when rooms were found to contain CRAB, the same cluster was found in multiple bed units 46 times (38.7%) suggesting it may have spread between them. On 70/119 occasions (58.8%), rooms adjacent to one another had the same CRAB cluster, suggesting that strain movement had potentially occurred (Table S6). Of the 116 occasions when complete sample sets were taken from different consecutive occupants of a single bed unit, the same CRAB clusters were found associated with consecutive patients on 16 occasions (13.8%; Table S7). The majority of potential strain movement between beds, rooms and consecutive patients could be solely accounted for by looking at environmental contamination (Tables S6 and S8). Six patients that were CRAB-negative within two days of admission later yielded CRAB-positive oral or rectal swabs. The CRAB clusters in these oral or rectal swabs had been present in their respective patients’ bed unit or room environments before the patients were admitted. These cases provide clear evidence for patient acquisition of CRAB from contaminated ICU environments.

### Plasmids were transmitted between CRAB clusters during the study

**Fig. 5:**
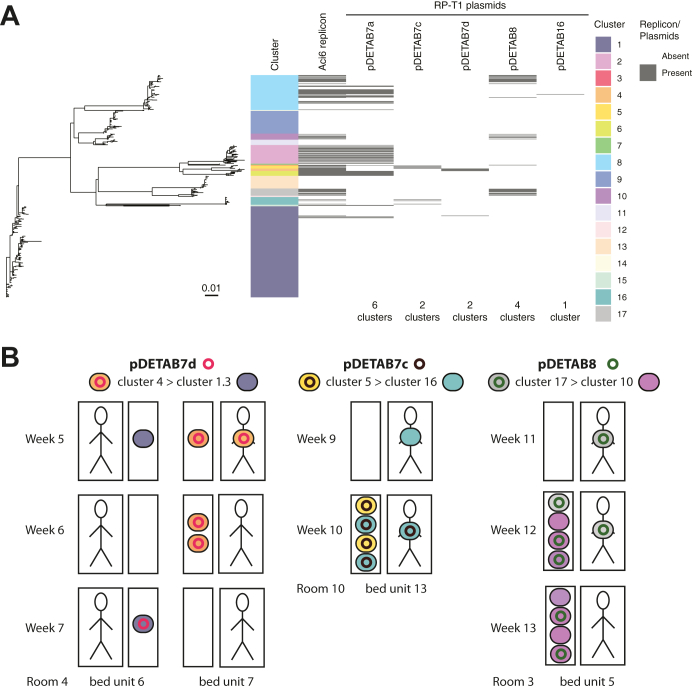
**Dissemination of RP-T1 plasmids amongst CRAB clusters.** A) Phylogenetic relationships of all GC2/ST187 isolates; tracks adjacent to the tips show isolate cluster designations, presence of the RP-T1 plasmid replicon, and the presence of specific RP-T1 plasmids determined from hybrid genome assemblies or detection of signature sequences in draft genomes. B) Schematic overview of putative pDETAB7d, pDETAB7c and pDETAB8 transfer events. Bed units are shown as paired boxes that represent patient-derived (larger boxes containing figurative person) and environmental (small boxes) sources of isolation. Weeks of isolation and room/bed unit numbers are labelled. Isolates are shown as coloured ovals and plasmids as coloured circles. The name of plasmids and isolate clusters, and the direction or putative plasmid transfer are indicated at the top of the panel. In all indicated transfer events the core genomes of putative recipient and transconjugants strains were identical and at least 50 kbp of the transferred RP-T1 plasmid backbones were identical between donors and transconjugants.

We detected eight plasmid replicon types in the CRAB collection (Table S9). R3-T4 (448 isolates), RP-T1 (126 isolates), R2-T1 (75 isolates), R3-T2 (19 isolates), and R3-T3 (3 isolates) replicons were found in the GC2/ST187 population (Fig. 1). R3-T4, R2-T1, and R3-T2 replicons were associated with conserved small plasmids (Table S9). Identical RP-T1 *rep* genes were found in multiple, disparate clusters (Fig. 1). The RP-T1 plasmids could be further differentiated into three plasmid backbone types, represented by pDETAB7 (with four variants distinguished by insertions or SNPs in an otherwise identical 72 kbp backbone), pDETAB8, and pDETAB16 (Figure S4; Table S9), which facilitated the analysis of their distributions amongst the CRAB population (Fig. 5A). We identified three putative transfer events, involving three different RP-T1 plasmids, six different CRAB clusters and three different rooms (Fig. 5B).

## Discussion

In this study, we used longitudinal sampling of an entire ICU, including staff, patients and the environment, to reveal the remarkable diversity of CRAB in this setting. The endemicity of CRAB, in a country where CRAB prevalence is reported to be highest globally,^29^ is in stark contrast to monoclonal outbreaks described in hospitals in low-prevalence high-income countries.^4^^,^^7^, ^8^, ^9^ We show that there was a large reservoir of CRAB in the hospital environment that was introduced to the ICU on multiple occasions in association with patient admissions. This environmental contamination appeared to drive dissemination within the ICU and a CRAB cluster in one room was often found in multiple bed units and adjacent rooms. Of 35 CRAB-positive patients in the study, 14 acquired CRAB during their ICU stay, and the acquired clusters were often found in the patient's immediate environment. It is clear from this study that without considering patient screening and environmental isolates it will be near-impossible to accurately assess complex polyclonal CRAB transmission pathways in ICUs. A similar observation, linking putative GC2 transmission clusters with environmental samples, was made in a recent study conducted in Vietnamese ICUs.^30^ We postulate that environmental surveillance in areas shown to be frequently contaminated, combined with rigorous patient screening, would present a more sensitive strategy for monitoring wards and beginning to reduce the CRAB burden in endemic settings.

Notably, as well as observing strain sharing between patients, we identified three putative plasmid transfer events between CRAB lineages. RP-T1 (formerly RepAci6) plasmids are known to carry antibiotic resistance genes, including carbapenemase genes,^31^^,^^32^ although the plasmids involved in transfer events in this study did not. In the laboratory, RP-T1 plasmids have been shown to be conjugative and to mediate the mobilisation of small antibiotic resistance plasmids.^32^ The transfer region of RP-T1 plasmids involved in transfer events here is complete, uninterrupted, and almost identical (1 SNP in 18,718 bp) to one that has been shown to be functional in laboratory experiments (GenBank accession KJ493819). Our observations provide the first evidence that RP-T1 plasmids transfer readily under hospital conditions in the absence of an obvious fitness advantage, suggesting that these plasmids can contribute to the ability of *A. baumannii* to acquire new traits in hospital settings.

Together these results highlight the important role of the environment in CRAB persistence and eventual acquisition by patients, and the need to target this reservoir with IPC measures, including through regular deep-cleaning of surfaces touched by patients and staff, isolation of patients known to carry CRAB, minimisation of patient relocation between beds, and enhanced staff hand-washing protocols.

## Contributors

ELD: data curation, formal analysis, visualisation, writing—original draft. HL: sample collection, sample processing, data curation, formal analysis. RAM: data curation, formal analysis, visualisation, writing—original draft, writing—review and editing. XH: supervision, formal analysis, project administration. XB: formal analysis. FG: sample collection. XC: sample collection. LZ: sample collection. MH: funding acquisition, conceptualisation, writing—review and editing. WvS: funding acquisition, conceptualisation, writing—review and editing. AM: funding acquisition, conceptualisation, formal analysis, writing—review and editing. YY: resources, funding acquisition, conceptualisation, writing—review and editing.

## Data sharing statement

Raw sequence data for all isolates is available via National Center for Biotechnology Information under BioProject accession PRJNA738868.

## Declaration of interests

The authors have no relevant conflicts of interest to declare.
